# Targeted imaging of very late antigen-4 for noninvasive assessment of lung inflammation-fibrosis axis

**DOI:** 10.1186/s13550-023-01006-0

**Published:** 2023-06-05

**Authors:** Qin Zhu, Clayton E. Barnes, Philip Z. Mannes, Joseph D. Latoche, Kathryn E. Day, Jessie R. Nedrow, Enrico M. Novelli, Carolyn J. Anderson, Sina Tavakoli

**Affiliations:** 1 Department of Radiology, University of Pittsburgh, UPMC Presbyterian Hospital, 200 Lothrop Street, Suite E200, Pittsburgh, PA 15213 USA; 2 Medical Scientist Training Program, University of Pittsburgh, Pittsburgh, PA USA; 3 Department of Medicine, University of Pittsburgh, Pittsburgh, PA USA; 4 Heart, Lung, Blood, and Vascular Medicine Institute, University of Pittsburgh Medical Center, Pittsburgh, PA USA; 5 Department of Chemistry, University of Missouri, Columbia, MO USA; 6 Department of Radiology, University of Missouri, Columbia, MO USA

**Keywords:** Lung fibrosis, Very late antigen-4, Molecular imaging, Inflammation, PET

## Abstract

**Background:**

The lack of noninvasive methods for assessment of dysregulated inflammation as a major driver of fibrosis (i.e., inflammation-fibrosis axis) has been a major challenge to precision management of fibrotic lung diseases. Here, we determined the potential of very late antigen-4 (VLA-4)-targeted positron emission tomography (PET) to detect inflammation in a mouse model of bleomycin-induced fibrotic lung injury.

**Method:**

Single time-point and longitudinal VLA-4-targeted PET was performed using a high-affinity peptidomimetic radiotracer, ^64^Cu-LLP2A, at weeks 1, 2, and 4 after bleomycin-induced (2.5 units/kg) lung injury in C57BL/6J mice. The severity of fibrosis was determined by measuring the hydroxyproline content of the lungs and expression of markers of extracellular matrix remodeling. Flow cytometry and histology was performed to determine VLA-4 expression across different leukocyte subsets and their spatial distribution.

**Results:**

Lung uptake of ^64^Cu-LLP2A was significantly elevated throughout different stages of the progression of bleomycin-induced injury. High lung uptake of ^64^Cu-LLP2A at week-1 post-bleomycin was a predictor of poor survival over the 4-week follow up, supporting the prognostic potential of ^64^Cu-LLP2A PET during the early stage of the disease. Additionally, the progressive increase in ^64^Cu-LLP2A uptake from week-1 to week-4 post-bleomycin correlated with the ultimate extent of lung fibrosis and ECM remodeling. Flow cytometry revealed that LLP2A binding was restricted to leukocytes. A combination of increased expression of VLA-4 by alveolar macrophages and accumulation of VLA-4-expressing interstitial and monocyte-derived macrophages as well as dendritic cells was noted in bleomycin-injured, compared to control, lungs. Histology confirmed the increased expression of VLA-4 in bleomycin-injured lungs, particularly in inflamed and fibrotic regions.

**Conclusions:**

VLA-4-targeted PET allows for assessment of the inflammation-fibrosis axis and prediction of disease progression in a murine model. The potential of ^64^Cu-LLP2A PET for assessment of the inflammation-fibrosis axis in human fibrotic lung diseases needs to be further investigated.

**Supplementary Information:**

The online version contains supplementary material available at 10.1186/s13550-023-01006-0.

## Background

Pulmonary fibrosis is a sequela of dysregulated tissue repair in a variety of diseases, including acute respiratory distress syndrome (ARDS), pneumonia, interstitial lung diseases (ILDs), occupational lung diseases, and iatrogenic causes, e.g., radiation, and medications [1, 2]. The etiological diversity of fibrotic lung diseases and heterogeneity of patients’ response to various treatments [3–5] urge for individualized management of patients based on noninvasive monitoring of cellular/molecular processes underlying the progression of maladaptive extracellular matrix (ECM) remodeling. Dysregulated inflammation is a major driver of adverse ECM remodeling (referred to as inflammation-fibrosis axis) across many fibrotic lung diseases [3–8]. Tissue injuries elicit a complex and highly coordinated inflammatory and repair response which often resolves upon the restoration of the homeostatic state [6]. However, continued and/or repetitive injuries (e.g., exposure to environmental hazards) or non-resolving inflammation (e.g., connective tissue diseases) may trigger sustained recruitment and activation of leukocytes and fibroblasts which lead to maladaptive ECM remodeling characterized by excessive deposition of collagen and other matrix proteins, architectural distortion, and ultimately functional impairments, such as impaired alveolar-capillary gas exchange [6].

Molecular imaging is a promising approach for noninvasive assessment of the inflammation-fibrosis axis. The initial attempts to image lung inflammation were focused on ^18^F-fluorodeoxyglucose (^18^F-FDG) positron emission tomography (PET) [9]. However, increased ^18^F-FDG uptake in inflamed tissues is nonspecific and reflects enhanced glucose utilization by a wide variety of cells involved in inflammation, and both physiological and dysregulated repair processes [9–11]. Considering its limited accuracy in characterizing the inflammation-fibrosis axis, ^18^F-FDG PET has not been broadly adopted as a precision medicine tool in inflammatory or fibrotic lung diseases [9]. Recent efforts have led to the development of novel tracers that target more specific aspects of the inflammation-fibrosis axis with promising results in pre-clinical and first-in-human studies. For example, collagen-targeted tracers can detect increased fibrosis burden within the lungs [12, 13]. However, this approach targets a mostly irreversible process in the pathogenesis of lung fibrosis (i.e., deposition of collagen) and may have limited utility for early monitoring of the therapeutic response. Alternatively, detection of fibroblast activation [14, 15] or dysregulated inflammation, as key drivers of maladaptive ECM remodeling, may overcome this limitation, and may be particularly advantageous in guiding immunomodulatory interventions. For example, molecular imaging of C–C chemokine receptor 2 (CCR2) by ^64^Cu-DOTA-ECL1i detects monocyte recruitment in murine models of acute lung injury (ALI) [16] and lung fibrosis [17] and in patients with idiopathic pulmonary fibrosis (IPF) [17]. Recently, we have shown that targeted imaging of chemokine-like receptor-1 allows for the detection of monocyte-derived macrophages in a mouse model of ALI [18]. Lung inflammation has also been successfully detected by targeting folate receptor-β [19]. The specificity of CCR2- and folate receptor-β-targeted imaging approaches is particularly valuable for the detection of monocyte/macrophage-driven lung inflammation. However, there is an unmet need for the development and validation of tracers targeting other leukocytes and inflammatory pathways contributing to lung inflammation.

Very late antigen-4 (VLA-4) (ɑ_4_β_1_ integrin) is highly expressed by leukocytes and plays central roles in their recruitment to inflamed tissues across different organs, including in various inflammatory lung diseases, such as ALI/ARDS [20–23], pneumonia [24–28], sepsis [29] and asthma [30–32]. Increased expression of VLA-4 ligands, i.e., vascular cell adhesion molecule-1 (VCAM-1) and fibronectin, have also been reported in fibrotic lung diseases [33–38], supporting the promise of VLA-4 as a biomarker of lung inflammation. We have recently shown that VLA-4-targeted PET using ^64^Cu-CB-TE1A1P-PEG4-LLP2A (^64^Cu-LLP2A), a peptidomimetic tracer with a high affinity to the active conformation of VLA-4, allows for quantitative detection of acute lung inflammation in a murine model of lipopolysaccharide-induced ALI [20]. Despite extensive investigations of this tracer in hematological and oncological [39–44] diseases, its utility in the assessment of lung inflammation-fibrosis axis has remained unexplored. In this study, we demonstrated the potential of VLA-4-targeted PET using ^64^Cu-LLP2A to detect ongoing lung inflammation and predict the risk of disease progression in a murine model of bleomycin-induced fibrotic injury. We also characterized VLA-4 expression across different leukocyte subsets to unravel the biological basis of increased ^64^Cu-LLP2A uptake in fibrotic lung injury.

## Methods

### Chemicals and reagents

The major commercially available reagents used in this study are listed in Additional file 1: Tables S1-S3.

### Mouse model of fibrotic lung injury

Animal studies were performed in accordance with a research protocol approved by the University of Pittsburgh Institutional Animal Care and Use Committee. Fibrotic lung injury was induced by intratracheal instillation of a single dose of bleomycin (2.5 U/kg in 60 µL phosphate-buffered saline (PBS) to 8–10-week-old C57BL/6 J mice (Jackson Laboratory, strain # 000664). Control mice were administered with 60 µL of sterile PBS intratracheally.

### Synthesis of CB-TE1A1P-PEG_4_-LLP2A, LLP2A-Biotin and LLP2A-Cy3

CB-TE1A1P-PEG_4_-LLP2A [44] and LLP2A-Biotin [45] were synthesized by Auspep (Tullamarine, Australia) and their purity and identity were confirmed by high-performance liquid chromatography (HPLC) and mass spectrometry (Additional file 1: Figs. S1-S2). The synthesis and characterization of LLP2A-Cy3 were previously reported [39, 46].

### Radiolabeling of CB-TE1A1P-PEG_4_-LLP2A

Radiolabeling of CB-TE1A1P-PEG_4_-LLP2A was performed by adding ~ 57.2 MBq ^64^CuCl_2_ (in 0.1 M HCl, obtained from Washington University or University of Wisconsin) per 1.0 nmol of CB-TE1A1P-PEG_4_-LLP2A in ammonium acetate buffer (0.5 M, containing 0.4 mM gentisic acid, pH = 6.5), as described previously [20, 41]. Reactions were allowed to proceed for ~ 30 min at 70 °C prior to verifying the radiochemical purity by radio-high-performance liquid chromatography (Additional file 1: Fig. S1D and Table S4). A threshold of radiochemical purity > 95% was set for the use of ^64^Cu-LLP2A in PET/CT experiments.

### ^64^Cu-LLP2A PET/CT and quantification of tracer uptake

Mice were injected with ^64^Cu-LLP2A (7.7 ± 0.2 MBq) via tail vein. PET/CT was performed ~ 4 h and 22 ± 2 h after ^64^Cu-LLP2A injection in three cohorts of mice. Cohort 1: Mice that underwent single time-point PET/CT at 2 weeks after intratracheal instillation of bleomycin (N = 3 males and 3 females) or PBS (N = 3 males and 3 females). Four mice (N = 2 males and 2 females) in the bleomycin-induced injury group from this cohort were co-injected with ~ 50 µg unlabeled LLP2A-CB-TE1A1P along with ^64^Cu-LLP2A to determine the in vivo specificity of the tracer uptake. Cohort 2: Mice that underwent longitudinal PET/CT at baseline as well as 1, 2, and 4 weeks after bleomycin administration (N = 4 males and 2 female). Cohort 3: Mice that underwent longitudinal PET/CT at 1 and 4 weeks after bleomycin administration (N = 8 males and 8 females). The image acquisition protocol consisted of static PET and non-contrast CT (Inveon, Siemens) according to our previous report [20]. PET and CT revealed no evidence of lung inflammation after intratracheal administration of PBS. Thus, data from baseline and PBS-injected mice were pooled as the control group.

Quantification of tracer uptake was performed by drawing regions of interest over the left and right lungs on co-registered PET and CT images (IRW software). Mean and maximal percent injected dose per mL of tissue (%ID/mL_mean_ and %ID/mL_max_) were quantified for each lung to determine global ^64^Cu-LLP2A uptake throughout the lungs and the uptake in the most inflamed regions, respectively.

Biodistribution of ^64^Cu-LLP2A was also determined after completion of the delayed PET/CT by ex vivo γ-counting (Wizard^2^, PerkinElmer) of the lungs and other major organs. Decay-corrected data are reported as the percentage of injected dose per gram of tissue (%ID/g).

### Gene expression assays

The lungs of the mice that underwent PET/CT were frozen after the completion of γ-counting. Right lungs were used for RNA extraction and cDNA synthesis, using Trizol and QuantiTect Reverse Transcription Kit, according to manufacturers’ protocols. mRNA expressions were quantified using TaqMan® primers (Additional file 1: Table S3) and a QuantStudio™3 real-time PCR system (Applied Biosystems). Transcript levels were normalized to the average expression levels of five housekeeping genes, 18S ribosomal RNA (*Rn18s*), hypoxanthine guanine phosphoribosyltransferase (*Hprt*), TATA box binding protein (*Tbp*), glucuronidase beta (*Gusb*), and glyceraldehyde-3-phosphate dehydrogenase (*Gapdh*).

### Quantification of the hydroxyproline content of the lungs

The hydroxyproline content of the lungs was measured with a colorimetric assay according to the manufacturer’s instructions with minor modifications. Absorbance was measured at 540 nm using a Biotek Synergy H4 multi-well spectrophotometer.

### Flow cytometric quantification of LLP2A binding to lung leukocytes

LLP2A-Biotin, an analog of CB-TE1A1P-PEG_4_-LLP2A, was synthesized by Auspep (Tullamarine, VIC, Australia) to serve as a surrogate to quantify ^64^Cu-LLP2A binding to various lung cells using flow cytometry.

Lungs of control and bleomycin-treated mice (5 males and 6 females, 1 week after intratracheal instillation of PBS vs. bleomycin) were harvested after euthanasia and perfusion of the pulmonary circulation with PBS. Lungs were minced into small pieces and dissociated into single cell suspension by incubation with 2 mg/mL collagenase D and 100 U/mL DNase I in PBS at 37 °C. After passing the cell suspensions through 70-µm cell strainers, red blood cells (RBC) were lysed. Cells were then counted, split between an appropriate number of tubes, and resuspended in HBSS buffer (containing Ca^2+^ and Mg^2+^) with 1% bovine serum albumin (BSA). Cells were then incubated with LLP2A-Biotin (50 nM) at room temperature for 15 min. Preincubation with 5 µM LLP2A-CB-TE1A1P, as a competitive blocker, was performed to quantify the specific binding to VLA-4 (specific binding of LLP2A-Biotin = binding in the absence of blocker—binding in the presence of blocker). Cells were then washed with HBSS buffer, and Fc receptors were blocked by adding anti-mouse CD16/CD32 antibody (Fc Block). Subsequently, a mixture of antibodies (Additional file 1: Table S2) was added to the samples for immunophenotyping of lung cells along with streptavidin-PE for the detection of LLP2A-Biotin binding. After 10 min incubation at room temperature, cells were washed with HBSS buffer and fixed with 4% formalin for 30 min at room temperature. Flow cytometry was performed using a LSR II Flow Cytometry (BD Biosciences) and analyzed using FlowJo software version 10.7.2 (BD Biosciences).

### Histology of murine lungs

Control and bleomycin-treated mice (4 males and 2 females) were euthanized and their pulmonary circulations were perfused with PBS. Lungs were then inflated by intratracheal instillation of ~ 1 mL optimal cutting temperature (OCT) compound and embedded in OCT. LLP2A-Cy3 histology was performed according to a reported protocol with minor modifications [42]. 10-µm cryosections were cut (Leica-CM1860). Permeabilization and blocking of nonspecific bindings were performed by 30-min incubation with 0.1% Triton X-100 and 10% bovine serum albumin in HBSS buffer at room temperature. Tissues were then incubated for 1 h at room temperature with 2 nM LLP2A-Cy3 in HBSS containing 1 mM MnCl_2_. The specificity of LLP2A-Cy3 binding was confirmed by pre- and co-incubation of tissues with 5 µM LLP2A-CB-TE1A1P. Tissues were then washed with HBSS buffer (containing 1 mM MnCl_2_) prior to 10-min incubation with 10 mM CuSO_4_ (pH: 5) at room temperature and final wash. Slides were then mounted with ProLong Gold Antifade Mountant containing DAPI. Fluorescent images were obtained using an Axio Vert microscope (Zeiss).

### Statistics

Statistical analysis was performed using Prism-9 (GraphPad). Data are presented as mean ± SEM. One-way analysis of variance followed by Fisher's Exact post hoc test was used to compare mean values of variables in > 2 groups. Non-paired t-test was used in flow cytometry experiments to compare the abundance of different leukocyte subsets and their mean fluorescent intensity between control and bleomycin-injured lungs. Correlation analyses were performed by Pearson’s test. Kaplan–Meier survival analysis and receiver operating characteristic curves were used to assess the potential of early PET in prediction of fatal disease (defined as spontaneous death or euthanasia as a humane endpoint due to weight loss > 25%). *P* < 0.05 was considered statistically significant.

## Results

### Increased uptake of ^64^Cu-LLP2A throughout different stages of fibrotic lung injury

**Fig. 1.**
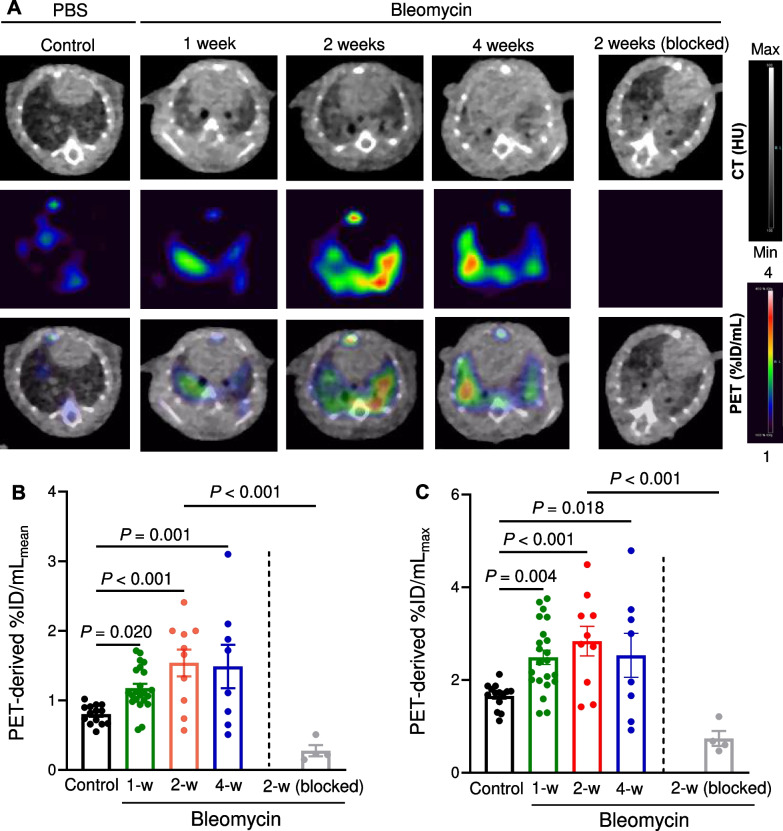
^64^Cu-LLP2A PET/CT in bleomycin-induced lung injury. Representative axial CT, PET, and co-registered PET/CT (**A**) of control versus bleomycin-treated mice acquired ~ 4 h after ^64^Cu-LLP2A administration demonstrate significant increases in the tracer uptake during different stages of fibrotic lung injury. The specificity of tracer uptake is confirmed by blocking ^64^Cu-LLP2A uptake, despite the presence of CT evidence of lung injury, by co-injection of excess non-labeled LLP2A (right panels in **A**). PET-derived quantification, measured as %ID/mL_mean_ (**B**) and %ID/mL_max_ (**C**), confirms significant increases in ^64^Cu-LLP2A uptake throughout different stages of the progression of bleomycin-induced lung injury as well its near-complete blockade by co-injection of non-labeled LLP2A. N = 14 (control), 22 (1-week), 10 (2-week), 8 (4-week), and 4 (2-week blocked). PBS = phosphate-buffered saline

PET/CT at ~ 4 h after ^64^Cu-LLP2A injection demonstrated intense tracer uptake at 1, 2, and 4 weeks after induction of fibrotic injury which was predominantly localized to high-attenuation regions in a peribronchial distribution as detected by CT (Fig. 1A). Coadministration of excess unlabeled LLP2A blocked the lung uptake of ^64^Cu-LLP2A in bleomycin-treated mice, confirming the specificity of the tracer uptake (Fig. 1A). Delayed PET/CT performed one day after ^64^Cu-LLP2A injection revealed a similar, though less intense, pattern of ^64^Cu-LLP2A uptake (Additional file 1: Fig. S3). Thus, quantitative analyses of ^64^Cu-LLP2A uptake in the rest of the study were performed with 4-h PET/CT images.

Two PET-derived measures of tracer uptake were assessed, maximal and mean percent of injected dose/mL of tissue (%ID/mL_max_ and %ID/mL_mean_), as the estimates for tracer uptake in the most-diseased region and global tracer uptake in the lungs, respectively. PET-derived quantification of ^64^Cu-LLP2A uptake demonstrated 46%, 92%, and 86% increase in the %ID/mL_mean_ (Fig. 1B) and 50%, 71% and 53% increase in the %ID/mL_max_ (Fig. 1C) at 1, 2, and 4 weeks after bleomycin administration compared to control mice. The in vivo specificity of ^64^Cu-LLP2A uptake was quantitatively confirmed by ~ 80% reduction in the tracer uptake in the lungs of mice co-injected with tracer and excess unlabeled LLP2A.

**Fig. 2:**
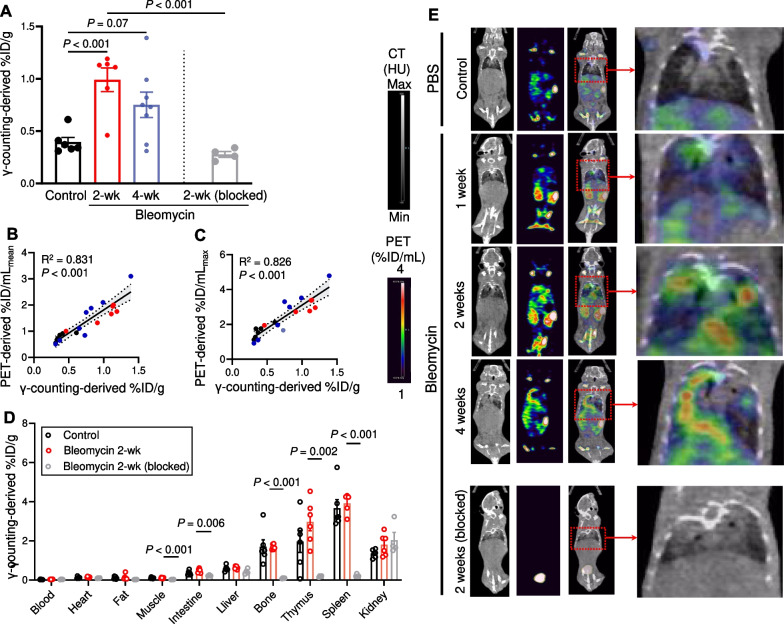
Biodistribution of ^64^Cu-LLP2A. Ex vivo γ-counting (**A**) confirms increased lung uptake of ^64^Cu-LLP2A at 2 and 4 weeks after bleomycin-induced fibrotic injury, which is blocked by co-injection of excess unlabeled LLP2A (ex vivo γ-counting was not performed at 1-week post-bleomycin as this timepoint was only assessed as part of the longitudinal PET/CT experiments). There are robust correlations between PET-derived (**B** ID/mL_mean_ and **C** %ID/mL_max_) and γ-counting-derived measures of ^64^Cu-LLP2A uptake, confirming the accuracy of noninvasive quantification of tracer uptake. γ-counting (**D**) and the visual assessment of whole-body PET (**E**) images demonstrate specific uptake of ^64^Cu-LLP2A in organs rich in VLA-4-expressing cells, including spleen, thymus, and bone marrow. However, ^64^Cu-LLP2A uptake in these organs is not significantly affected by bleomycin administration. N = 6 (control), 6 (2-week), 8 (4-week), and 4 (2-week blocked)

Ex vivo γ-counting of harvested lungs, the gold standard method for quantification of tracer uptake, demonstrated a similar pattern of increased ^64^Cu-LLP2A uptake in bleomycin-injured lungs (Fig. 2A). Notably, PET-derived (i.e., %ID/mL_mean_ and %ID/mL_max_) and γ-counting-derived (%ID/g) measures of ^64^Cu-LLP2A uptake strongly correlated (Fig. 2B, C), confirming the accuracy of in vivo quantification of tracer uptake.γ-counting (Fig. 2D) and whole-body PET (Fig. 2E) assessment of biodistribution revealed a significant uptake of ^64^Cu-LLP2A in multiple organs rich in immune cells, including spleen, thymus, and bone.

### Prediction of progressive fibrotic lung injury by ^64^Cu-LLP2A PET

**Fig. 3:**
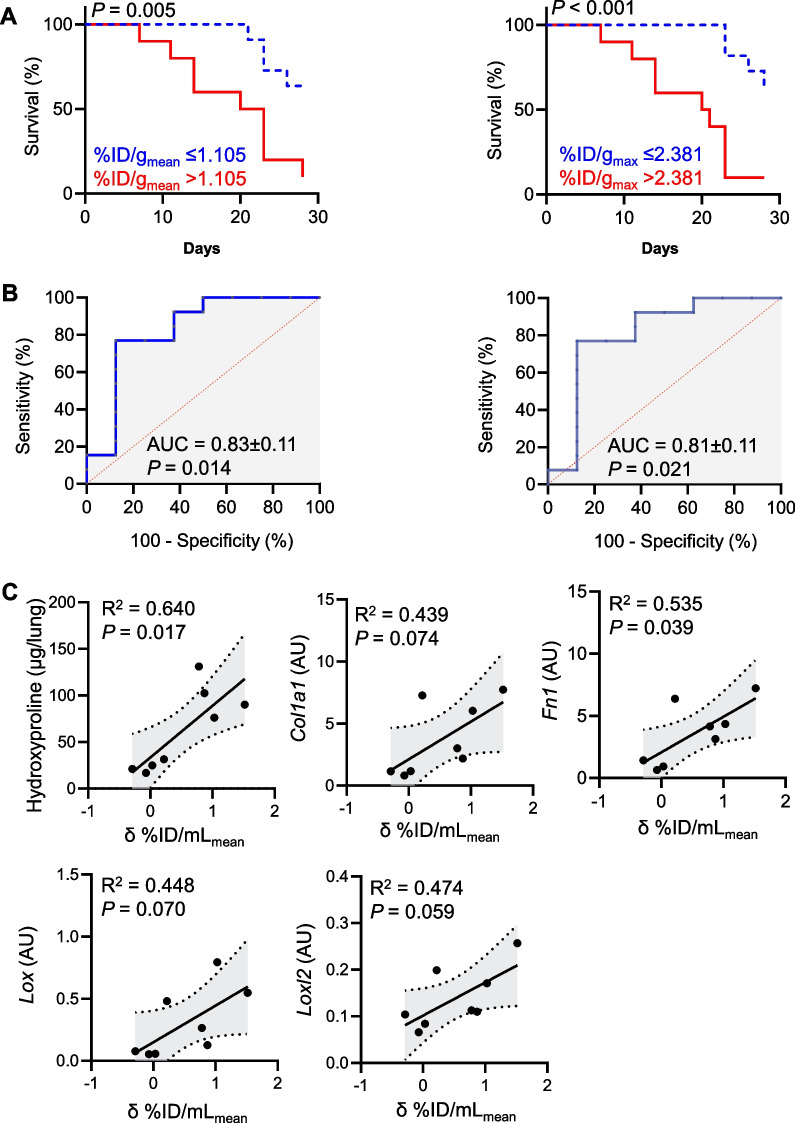
Prognostication of bleomycin-induced lung injury by ^64^Cu-LLP2A PET. High lung uptake of ^64^Cu-LLP2A at 1-week post-bleomycin, defined as the uptake above the median %ID/mL_mean_ (left panel) or %ID/mL_max_ (right panel), predicts a significantly worse survival over the course of 4 weeks (**A**). Similarly, both %ID/mL_mean_ (left panel) and %ID/mL_max_ (right panel) of ^64^Cu-LLP2A uptake at 1-week post-bleomycin strongly predict fatal lung injury as assessed by ROC curve analysis (**B**). The progressive increase in lung uptake of ^64^Cu-LLP2A from week-1 to week-4 post-bleomycin correlates with the severity of fibrosis and ECM remodeling as determined by the hydroxyproline content of the lungs and mRNA expression of markers of matrix remodeling at week-4, including collagen 1 (*Col1a1*), fibronectin (*Fn1*), lysyl oxidase (*Lox*) and lysyl oxidase-like 2 (*Loxl2*) (**C**). N = 21 for panels **A** and **B**, N = 8 for panel **C**

To determine the prognostic potential of VLA-4-targeted PET during the early pneumonitis phase of bleomycin-induced lung injury, we performed a survival analysis of mice based on their ^64^Cu-LLP2A uptake at week 1 post-bleomycin. Mice with lung uptake above the median %ID/mL_mean_ and %ID/mL_max_ had a significantly worse survival compared to those with uptake values at or below the median (Fig. 3A). Receiver operating characteristic (ROC) curve analyses to assess the accuracy of early ^64^Cu-LLP2A PET in predicting fatal fibrotic lung injury demonstrated excellent performance of both %ID/mL_mean_ and %ID/mL_max_ of ^64^Cu-LLP2A uptake at one-week post-bleomycin in predicting fatal fibrotic lung injury with areas under the curve of 0.83 ± 0.11 and 0.81 ± 0.11 (Fig. 3B). Additionally, the progressive increase in ^64^Cu-LLP2A uptake from week-1 to week-4 post-bleomycin correlated with the extent of lung fibrosis and ECM remodeling at week-4 post-bleomycin among the survivors of the longitudinal studies as estimated by the hydroxyproline content of the lungs and the mRNA expression of markers of ECM remodeling, including *Col1a1*, *Fn1*, *Lox*, and *Loxl2* (Fig. 3C).

### Increased expression and accumulation of VLA-4-expressing leukocytes in fibrotic lung injury

**Fig. 4:**
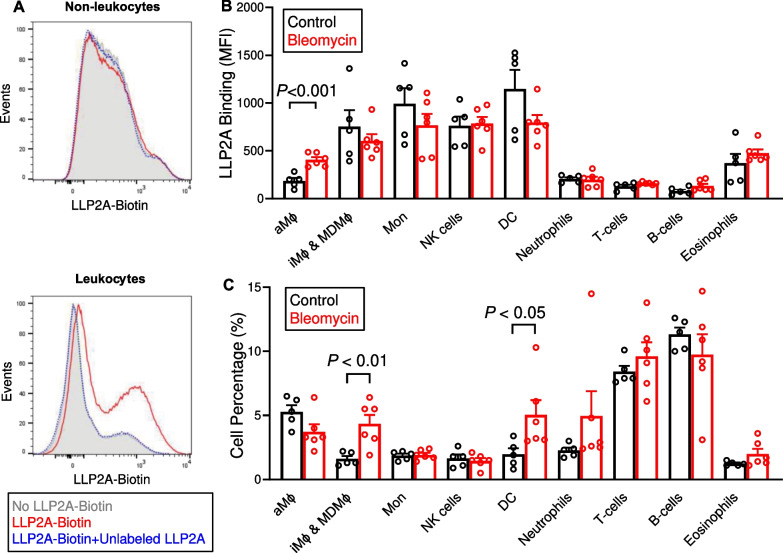
Flow cytometric quantification of LLP2A binding to different lung leukocytes. Representative histograms from flow cytometry of enzymatically dissociated murine lung single cells (**A**) demonstrate negligible binding of LLP2A-Biotin to CD45-negative cells (top panel) while different levels of binding to LLP2A-Biotin are present in CD45-positive leukocytes (bottom panel). The specificity of LLP2A-Biotin binding is confirmed by near-complete blocking of its binding in cells co-incubated with excess non-fluorescent LLP2A. Cell-type specific analysis of the flow cytometry data demonstrated that LLP2A-Biotin binding nearly doubles in alveolar macrophage (aMφ) of bleomycin-injured lungs compared to control lungs, while is not significantly different compared to control lungs in the remaining leukocyte subsets (**B**). There is a significant increase in the abundance of interstitial and monocyte-derived macrophage (iMφ & MDMφ) as well as dendritic cells (DC) at 1-week post-bleomycin injury versus controls (**C**). N = 5 (control group) and 6 (bleomycin group)

To estimate the contribution of different cell types to ^64^Cu-LLP2A uptake, we quantified the binding of LLP2A-Biotin to enzymatically dissociated lung single cells using flow cytometry (gating strategy provided in Additional file 1: Fig. S4). Notably, LLP2A-Biotin binding was mostly restricted to leukocytes with negligible binding to CD45-negative cells (Fig. 4A). LLP2A-Biotin binding was markedly higher in macrophages, monocytes, dendritic cells, and natural killer cells compared to other leukocyte subsets, i.e., lymphocytes, neutrophils, and eosinophils (Fig. 4B). Interestingly, LLP2A-Biotin binding to alveolar macrophages nearly doubled in bleomycin-injured lungs compared to control lungs, while it was unchanged in the remaining leukocyte subsets. Bleomycin also led to a significant increase in the abundance of VLA-4-expressing interstitial and monocyte-derived macrophages as well as dendritic cells in the lungs (Fig. 4C). Together, these data suggest that increased ^64^Cu-LLP2A uptake in bleomycin-induced lung injury is driven by a combination of increased expression of VLA-4 by alveolar macrophages and accumulation of VLA-4-expressing interstitial and monocyte derived macrophages as well as dendritic cells.

### Increased VLA-4-expression in inflamed and fibrotic regions of lungs

**Fig. 5:**
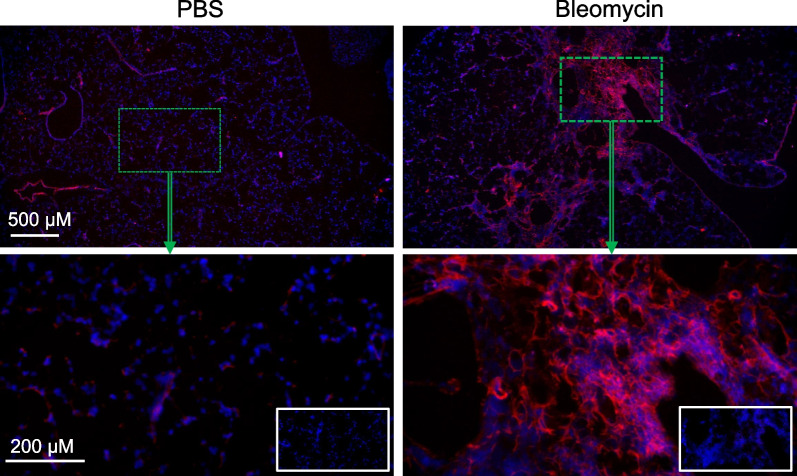
Histological assessment of VLA-4 expression in murine lungs with bleomycin-induced fibrosis. Representative low-magnification (top panels) and high-magnification (bottom panels) images demonstrate a marked increase in the binding of LLP2A-Cy3 (red) in fibrotic lungs of the mice at 4 weeks after bleomycin administration compared to the control lungs. The specificity of LLP2A-Cy3 binding is confirmed by the blocking of LLP2A-Cy3 signal in tissues co-incubated with excess non-fluorescent LLP2A (the insets in the right lower corners of high-magnification images). Tissues are counterstained with DAPI nuclear staining (blue). N = 3 per group

Histological assessment of murine lungs using LLP2A-Cy3 demonstrated a marked increase in VLA-4 expression in bleomycin-injured lungs (Fig. 5) which was mostly localized to fibrotic and inflammatory regions with a peribronchial-predominant distribution pattern, as expected with the intra-tracheal delivery route of bleomycin administration. The specificity of staining was confirmed by blocking of the LLP2A-Cy3 signal in tissues co-incubated with non-fluorescent LLP2A.

## Discussion

This study demonstrates the promise of ^64^Cu-LLP2A PET to detect lung inflammation at different stages of bleomycin-induced fibrotic injury and predict the risk of disease progression. We also showed that VLA-4 expression is almost entirely restricted to leukocytes. Additionally, our flow cytometry data suggest that increased ^64^Cu-LLP2A uptake in bleomycin-injured lungs was primarily driven by increased VLA-4 expression by alveolar macrophages along with accumulation of VLA-4-expressing interstitial and monocyte-derived macrophages as well as dendritic cells.

Noninvasive imaging of inflammation, as a major driver of dysregulated ECM remodeling, is an emerging precision medicine approach in fibrotic lung diseases. Considering the complexities of the inflammation-fibrosis axis, different processes have been targeted to depict different aspects of the dysregulated immune response in fibrotic lung diseases [9], an effort that mirrors the endeavor to develop and optimize various immunomodulatory and anti-fibrotic drugs for these diseases. Tracers targeting fibroblast activation protein [14, 15], collagen [12, 13], CCR2 [47], and folate receptor-β [19] are promising examples that have been explored in preclinical and small clinical studies and depict different and complementary aspects of the dysregulated inflammation-fibrosis axis, e.g., fibroblast activation, increased tissue fibrosis burden, monocyte recruitment, and macrophage activation. Considering the key roles of VLA-4 in leukocytes’ recruitment to inflamed tissues and its high expression by various immune cells [22–26, 31, 32, 48, 49], we sought to determine if VLA-4-targeting allows for assessment of the inflammation-fibrosis axis in a murine model of bleomycin-induced fibrotic injury.

^64^Cu-LLP2A is a high-affinity VLA-4-targeting tracer with an established safety profile and favorable kinetics in first-in-human studies [50] and that is on the verge of clinical translation in at least two clinical conditions, i.e., multiple myeloma [Clinical Trial # NCT03804424] [50] and sickle cell disease [Clinical Trial # NCT04925492]. However, the application of ^64^Cu-LLP2A PET in inflammatory diseases has been limited to a few studies [20, 46]. For example, ^64^Cu-LLP2A uptake has been shown in lung granulomas in a nonhuman primate model of tuberculosis with a pattern that was distinct compared to ^18^F-FDG uptake and significantly correlated with the number of macrophage and T cells within the granulomas [46]. We have also recently reported that lung uptake of ^64^Cu-LLP2A is significantly increased in a murine model of lipopolysaccharide-induced ALI and correlated with the expression of multiple markers of acute inflammation [20]. Here, we extended the scope of our previous study by assessing the potential of ^64^Cu-LLP2A PET in imaging sustained lung inflammation and prognostication of disease severity in a murine model of bleomycin-induced fibrotic injury. Our data demonstrated an increased lung uptake of ^64^Cu-LLP2A throughout the progression of fibrotic injury as assessed by imaging at 1, 2 and 4 weeks after administration of bleomycin. Strong correlations were present between PET-derived and γ-counting-derived measures of ^64^Cu-LLP2A uptake, confirming the accuracy of in vivo quantification of tracer uptake. Notably, high uptake of ^64^Cu-LLP2A 1 week after bleomycin administration was associated with a significantly worse survival, and progressive increase in ^64^Cu-LLP2A from week-1 to week-4 post-bleomycin correlated with higher contents of lung hydroxyproline and expression of ECM remodeling markers, underscoring the potential of ^64^Cu-LLP2A in predicting the course of fibrotic lung injury in this model.

We next sought to determine the immunophenotype of cells contributing to ^64^Cu-LLP2A uptake. Although several prior studies have reported the accumulation of VLA-4-expressing leukocytes in inflammatory lung diseases by flow cytometry or immunostaining for α_4_ and β_1_ integrin subunits [21–32], a comprehensive immunophenotypic characterization of VLA-4-expressing cells in fibrotic lung injury has not been reported. Additionally, α_4_ and β_1_ integrins may be expressed by cells as components of integrins other than VLA-4. To address this limitation, we utilized LLP2A-Biotin as a specific ligand for active VLA-4 [45] and demonstrated by flow cytometry that VLA-4 expression is almost entirely restricted to leukocytes, confirming the utility of ^64^Cu-LLP2A for imaging lung inflammation. Consistent with the crucial role of VLA-4 in recruitment of various leukocyte subsets, LLP2A-Biotin binding was present across a broad range of leukocyte subsets, though it was higher in macrophages, monocytes, dendritic cells, and natural killer cells compared to neutrophils, lymphocytes, and eosinophils. Cell-specific comparison between control and bleomycin-injured lungs demonstrated that LLP2A-Biotin binding was significantly increased in alveolar macrophages of bleomycin-injured lungs compared to those of controls. While LLP2A-binding to other leukocyte subsets was comparable between control and injured lungs, bleomycin-induced injury led to a significant accumulation of VLA-4-expressing interstitial and monocyte-derived macrophages as well as dendritic cells, suggesting the contribution of these cells to the increased lung uptake of ^64^Cu-LLP2A in this model. Histology using a fluorescent analog of LLP2A confirmed a marked accumulation of VLA-4-expressing cells in bleomycin-induced injury which were mostly localized to the fibrotic regions.

A limitation of this study pertains to the animal model of fibrotic lung injury. Lung fibrosis occurs in a wide variety of diseases and the murine model of bleomycin-induced lung fibrosis does not completely recapitulate the complexities of these diseases. A notable example is that an early robust pneumonitis phase is a key feature of bleomycin-induced fibrotic lung injury, which does not adequately mimic fibrotic diseases with milder inflammation, e.g., IPF [51]. However, the bleomycin model remains the most extensively validated model of fibrotic lung injury across various species with established stages of acute pneumonitis proceeding to chronic pneumonitis and fibrosis, and it is particularly valuable for the assessment of the inflammation-fibrosis axis [17, 51] It is noteworthy that while the lack of beneficial effects from immunosuppression in patients with IPF [52, 53] has raised debates about the role of inflammation in this specific category of lung fibrosis [54], numerous findings (including high levels of proinflammatory cytokines, e.g., TNF-α and IL-8) support dysregulated innate and adaptive immune responses in IPF (reviewed in [4, 54]). Moreover, an extensive body of literature supports the crucial role of inflammation and the beneficial effects of immunomodulation in most other causes of lung fibrosis (reviewed in [6, 54–56]), including connective tissue diseases, sarcoidosis, hypersensitivity pneumonitis, and post-ARDS fibrosis. Another limitation of this study is that PET/CT acquisition and the measurement of tracer uptake were performed without respiratory-gating and air-fraction correction. Thus, PET-derived quantification may have overestimated the in vivo tracer uptake in fibrotic lungs. Nevertheless, the strong correlation between PET-derived and weight-corrected γ-counting-derived measures of tracer uptake supports the overall high accuracy of our in vivo quantification approach.

## Conclusion

This study demonstrates the potential of VLA-4-targeted PET in monitoring ongoing inflammation throughout different stages of fibrotic lung injury and predicting the risk of disease progression. Considering the established safety of ^64^Cu-LLP2A and its recent Investigational New Drug status by the US FDA in hematological/oncological diseases [50], the findings of our study may provide a new application for ^64^Cu-LLP2A in future translational studies for a variety of fibrotic lung diseases, including post-ARDS fibrosis which has been a major healthcare concern in the post-COVID-19 era.

## Supplementary Information

**Additional file 1: Fig. S1**. Chemical structure and radiolabeling of CB-TE1A1P-PEG4-LLP2A. **Fig. S2**. Confirmation of the purity and identity of LLP2A-Biotin. **Fig. S3**. Delayed ^64^Cu-LLP2A PET/CT in bleomycin-induced lung injury. **Fig. S4**. Flow cytometric gating strategy for identification of leukocyte subsets in murine lungs. **Table S1**. List of general reagents. **Table S2**. List of reagents used for flow cytometry. **Table S3**. List of primers used for quantitative RT-PCR. **Table S4**. Radio-HPLC methods.

## Data Availability

The datasets used and analyzed during the current study are available from the corresponding author upon reasonable request.

## Abbreviations

^18^F-FDG: ^18^F-fluorodeoxyglucose;
^64^Cu-LLP2A: ^64^Cu-CB-TE1A1P-PEG4-LLP2A
ALI: Acute lung injury
ARDS: Acute respiratory distress syndrome
CCR2: C–C chemokine receptor 2
*Col1a1*: Collagen type-1-ɑ1
ECM: Extracellular matrix
*Fn1*: Fibronectin-1
ILD: Interstitial lung disease
IPF: Idiopathic pulmonary fibrosis
*Lox*: Lysyl oxidase
*Loxl2*: Lysyl oxidase-like 2
PET: Positron emission tomography
VLA-4: Very late antigen-4
