# Supplementary material for: Targeted imaging of very late antigen-4 for noninvasive assessment of lung inflammation-fibrosis axis
Source: EJNMMI Res. 2023 Jun 5;13:55. doi: 10.1186/s13550-023-01006-0 (PMC10240482; doi:10.1186/s13550-023-01006-0)
Supplement: Supplementary file 1 — Additional file 1: Fig. S1. Chemical structure and radiolabeling of CB-TE1A1P-PEG4-LLP2A. Fig. S2. Confirmation of the purity and identity of LLP2A-Biotin. Fig. S3. Delayed 64Cu-LLP2A PET/CT in bleomycin-induced lung injury. Fig. S4. Flow cytometric gating strategy for identification of leukocyte subsets in murine lungs. Table S1. List of general reagents. Table S2. List of reagents used for flow cytometry. Table S3. List of primers used for quantitative RT-PCR. Table S4. Radio-HPLC methods. [file 13550_2023_1006_MOESM1_ESM.pdf]

SUPPLEMENTAL METHODS AND RESULTS

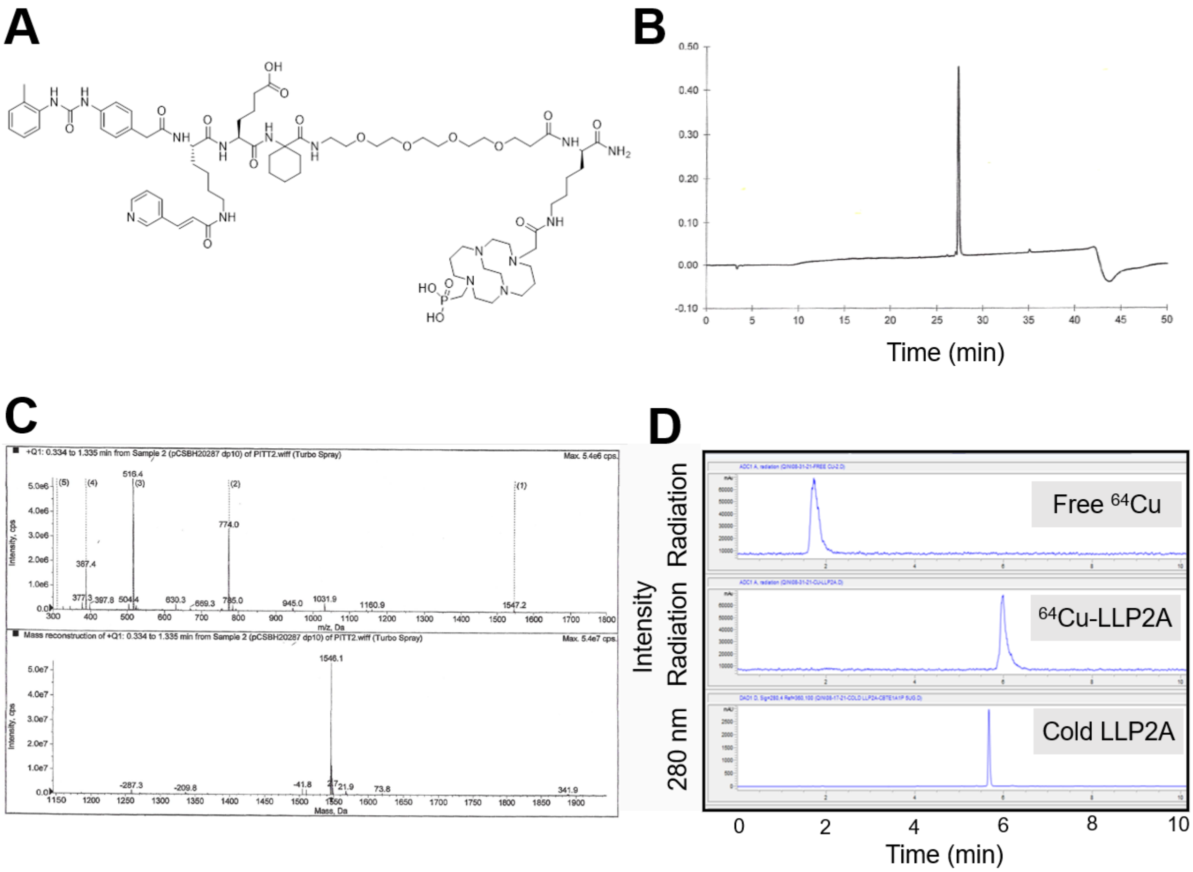

Fig. S1. Chemical structure and radiolabeling of CB-TE1A1P-PEG4-LLP2A. The chemical structure **(A)** of LLP2A-CB-TE1A1P (molecular weight: 1,547) which was synthesized by Auspep (Australia) and its purity and identity was confirmed by high-pressure liquid chromatography **(B)** and mass spectrometry **(C)**. Quality control of copper-64 radiolabeling of CB-TE1A1P-PEG4-LLP2A was performed by radio-HPLC to confirm radiolabeling efficiency >95% prior to injection of ^{64}Cu-LLP2A to animals. A representative example **(D)** demonstrates a retention time of ~2 minutes for free copper-64 (^{64}Cu) (top panel) *vs.* ~ 6 minutes for ^{64}Cu-LLP2A (middle panel) and unlabeled LLP2A (lower panel).

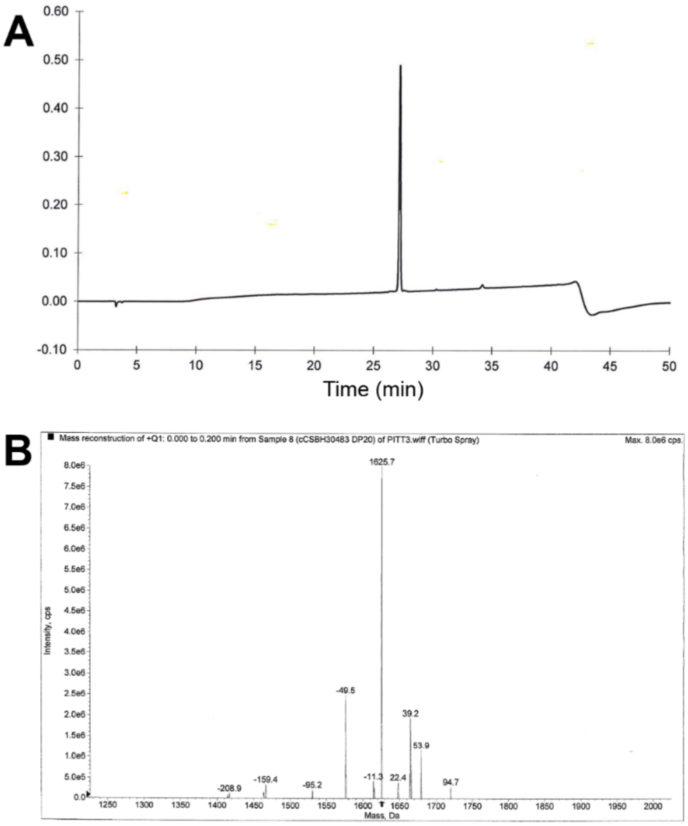

Fig. S2. Confirmation of the purity and identity of LLP2A-Biotin. LLP2A-Biotin (molecular weight: 1,626) was synthesized by Auspep (Australia) and its purity and identity was confirmed by high-pressure liquid chromatography **(A)** and mass spectrometry **(B)**.

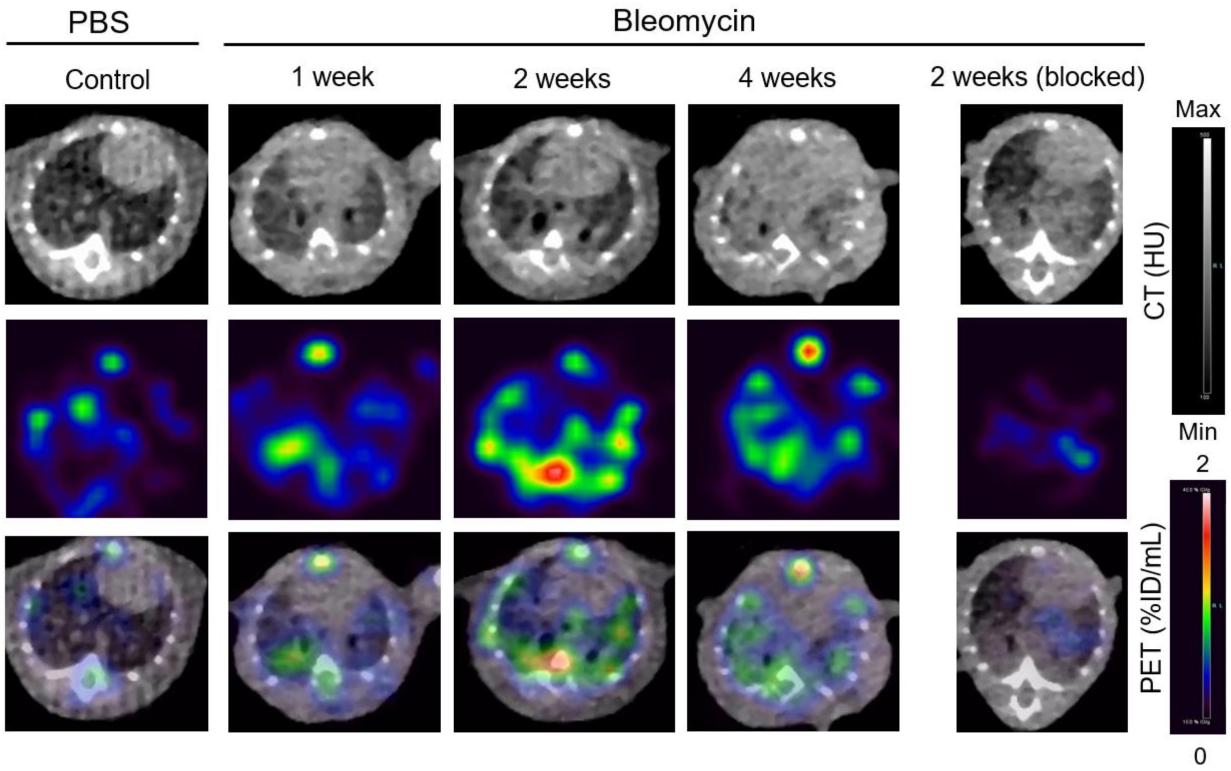

Fig. S3. Delayed ^{64}Cu-LLP2A PET/CT in bleomycin-induced lung injury. Representative axial CT, PET, and co-registered PET/CT acquired one day after administration of ^{64}Cu-LLP2A demonstrate persistent accumulation of the tracer in bleomycin-injured lungs compared to control lungs. The specificity of ^{64}Cu-LLP2A uptake is demonstrated by blocking ^{64}Cu-LLP2A uptake by co-injection of excess non-labeled LLP2A-CB-TE1A1P.

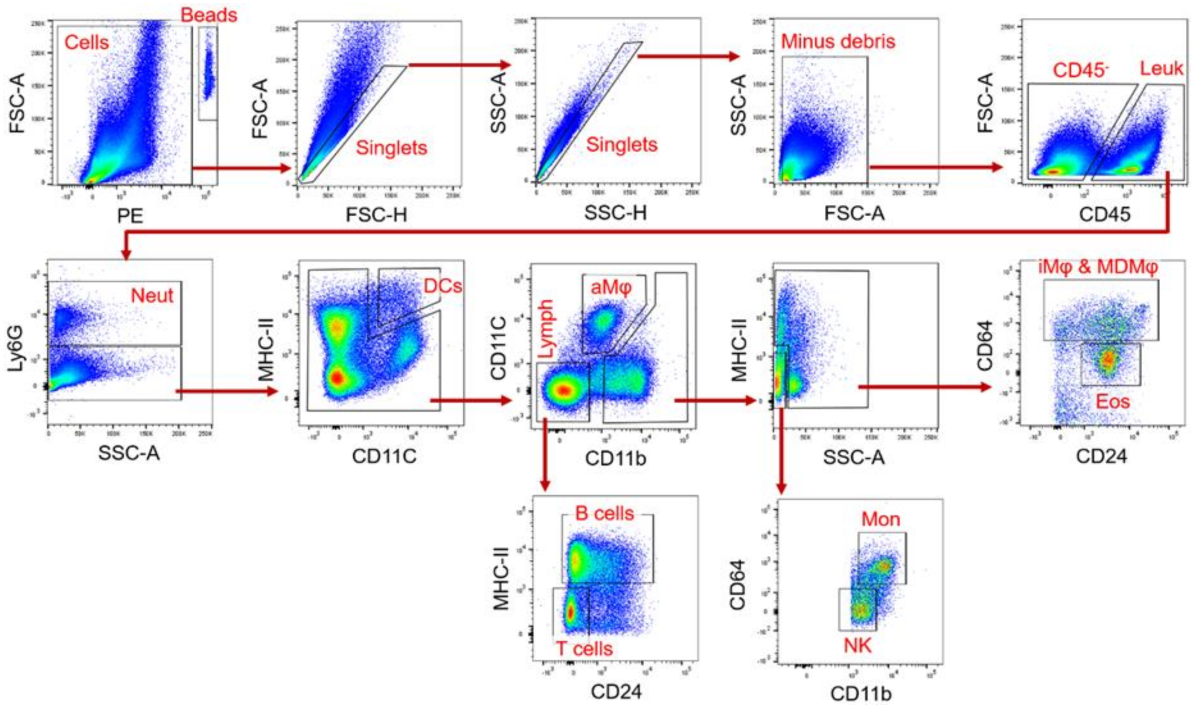

Fig. S4. Flow cytometric gating strategy for identification of leukocyte subsets in murine lungs. A representative gating strategy for identification of the major leukocyte subsets in enzymatically dissociated single cells from murine lungs. Abbreviations: aMφ: alveolar macrophages; DC: dendritic cells; MDMφ: monocyte-derived macrophages; Eos: eosinophils; iMφ: interstitial macrophages; Leuk: leukocytes; Lymph: lymphocytes; Mon: monocytes; Neut: neutrophils; NK: natural killer cells.

Table S1. List of general reagents.

Reagent	Company	Catalog #
Bleomycin sulfate	Sigma-Aldrich	B8416-15UN
Low protein binding tubes	Eppendorf	022431081
Cell strainer (70 µm)	VWR	10199-656
Collagenase D	Sigma	11088866001
DNase I	Sigma	D4527-10KU
HBSS with Ca^{2+} and Mg^{2+}	Gibco	14025-076
Hydroxyproline colorimetric assay kit	BioVision	K555
Phosphate-buffered saline (PBS)	Lonza	17-512F
ProLong Gold Antifade Mountant with DAPI	Thermo Fisher	P36931
RBC lysis buffer (10X)	BioLegend	420301
QuantiTect reverse transcription kit	Qiagen	205313
TaqMan gene expression master mix	Thermo Fisher	4369510
Triton X-100	Sigma	9002-93-1
TRIzol	Thermo Fisher	15596026

Table S2. List of reagents used for flow cytometry.

Antibody	Company	Catalog #
Anti-mouse/human CD11b-AF488	BioLegend	101217
Anti-mouse CD11c-PerCP	BioLegend	117326
Anti-mouse CD24-AF700	BioLegend	101836
Anti-mouse CD45-BV421	BioLegend	103134
Anti-mouse CD64-APC	BioLegend	139306
Anti-mouse Ly6C-APC-Cy7	BioLegend	128026
Anti-mouse Ly6G-BV395	BD Biosciences	563978
Anti-mouse I-A/I-E-BV605	BioLegend	107639
Anti-mouse SiglecF-BV510	BD Biosciences	740158
Anti-Mouse CD16/CD32 (Fc Block)	BD Pharmingen	553141
Precision count beads	BioLegend	424902
Streptavidin-Phycoerythrin	BioLegend	405203

Table S3. List of primers used for quantitative RT-PCR.

Assay Name	Gene	Assay ID
Rn18s	18S ribosomal RNA	Mm03928990_m1
Hprt	Hypoxanthine guanine phosphoribosyltransferase	Mm01545399_m1
Tbp	TATA box binding protein	Mm00446971_m1
Gusb	Glucuronidase, beta	Mm01197698_m1
Gapdh	Glyceraldehyde-3-phosphate dehydrogenase	Mm99999915_g1
Lox	Lysyl oxidase	Mm00495386_m1
Loxl2	Lysyl oxidase like-2	Mm00804740_m1
Col1a	Pro-alpha-1 chains of type I collagen	Mm00801666_g1
Fn1	Fibronectin-1	Mm01256744_m1

Table S4. Radio-HPLC methods (Agilent 1260 Infinity HPLC, C18 Luna Analytical Column).

**Time**	**Solvent 1:** H_2O with 0.1% v/v trifluoroacetic acid	**Solvent 2:** Acetonitrile with 0.1% v/v trifluoroacetic acid	**Flow rate (mL/min)**
0 → 2 min	100%	0%	2.0
2 → 8 min (gradient)	100% → 10%	0% → 90%	2.0
8 → 12 min	10%	90%	2.0
